# A switchable light-responsive azopolymer conjugating protein micropatterns with topography for mechanobiological studies

**DOI:** 10.3389/fbioe.2022.933410

**Published:** 2022-07-22

**Authors:** Chiara Cimmino, Paolo A. Netti, Maurizio Ventre

**Affiliations:** ^1^ Department of Chemical, Materials and Industrial Production Engineering, University of Naples Federico II, Naples, Italy; ^2^ Center for Advanced Biomaterials for Healthcare@CRIB, Fondazione Istituto Italiano di Tecnologia, Naples, Italy; ^3^ Interdisciplinary Research Centre on Biomaterials, University of Naples Federico II, Naples, Italy

**Keywords:** dynamic substrates, light-responsive, cell adhesion, mechanotransduction, cell shape, nucleus shape, azopolymer

## Abstract

Stem cell shape and mechanical properties *in vitro* can be directed by geometrically defined micropatterned adhesion substrates. However, conventional methods are limited by the fixed micropattern design, which cannot recapitulate the dynamic changes of the natural cell microenvironment. Current methods to fabricate dynamic platforms usually rely on complex chemical strategies or require specialized apparatuses. Also, with these methods, the integration of dynamic signals acting on different length scales is not straightforward, whereas, in some applications, it might be beneficial to act on both a microscale level, that is, cell shape, and a nanoscale level, that is, cell adhesions. Here, we exploited a confocal laser-based technique on a light-responsive azopolymer displaying micropatterns of adhesive islands. The laser light promotes a directed mass migration and the formation of submicrometric topographic relieves. Also, by changing the surface chemistry, the surfacing topography affects cell spreading and shape. This method enabled us to monitor in a non-invasive manner the dynamic changes in focal adhesions, cytoskeleton structures, and nucleus conformation that followed the changes in the adhesive characteristic of the substrate. Focal adhesions reconfigured after the surfacing of the topography, and the actin filaments reoriented to coalign with the newly formed adhesive island. Changes in cell morphology also affected nucleus shape, chromatin conformation, and cell mechanics with different timescales. The reported strategy can be used to investigate mechanotransduction-related events dynamically by controlling cell adhesion at cell shape and focal adhesion levels. The integrated technique enables achieving a submicrometric resolution in a facile and cost-effective manner.

## Introduction

Cell adhesion has a direct impact on focal adhesions (FAs) formation, cytoskeleton arrangement, cell contractility, and nucleus shape, which altogether affect several cell functions, including morphology, proliferation, migration, and differentiation (Ventre et al., 2012; Anderson et al., 2016). These are essential elements of mechanobiology that aims to investigate how cells integrate stimuli from the surrounding mechanical environment and convert them into biochemical events (Jansen et al., 2015). Mechanical stimuli are not limited to externally applied stresses and strains, but they also derive from cell-generated forces, which result from the adaptation of cell adhesion and contractility to the biophysical microenvironment (Eyckmans et al., 2011). For instance, by controlling cell shape *in vitro* through adhesive micropatterns, it is possible to alter FA morphology and cytoskeletal dynamics, eventually affecting cell fate through mechanotransduction pathways (McBeath et al., 2004; Kilian et al., 2010). Similarly, the topography and elasticity of the microenvironment also impact FA formation and contractility, thus, resulting in potent regulators of cell behaviour (Ventre et al., 2012; Ventre and Netti, 2016b). The cell recognition and response to the mechanical microenvironment are intrinsically dynamic phenomena, as cells are sensitive to spatiotemporal changes in the surrounding mechanical inputs. In particular, different doses of mechanical stimulation, in the form of time-changes in the stiffness or topography of the microenvironment, produce very different cell responses, including diverse differentiation capabilities (Yang et al., 2014; Rammensee et al., 2017). Notwithstanding this, the majority of the studies aimed at characterising cell response to the biophysical and mechanical microenvironment have been undertaken under static conditions. In this contest, the substrates exhibit patterns of signals fixed in space and time, thus, providing constant mechanical stimulation to cells.

Advancements in materials engineering and micro- and nano-fabrication technologies have recently enabled the development of culturing platforms able to display patterns of biochemical and biophysical signals whose features change in time and space according to selected programmes. These developments have led to a variety of approaches in which material properties such as surface chemistry and mechanical or topographical properties can be dynamically modified in response to user-directed stimuli, including temperature changes, light irradiation, and electromagnetic fields (Roy et al., 2010; Stuart et al., 2010; Uto et al., 2017). Many dynamic platforms were engineered with the purpose of enabling/disabling the adsorption of adhesive proteins by changing specific physical surface properties such as hydrophobicity, wettability, and surface charge, or altering the chemical composition of the surface (Cimmino et al., 2018). Other platforms, typically hydrogels, were synthesized to display time-varying stiffness, and these usually rely on polymer degradation or crosslinking that can either proceed spontaneously or can be activated by external triggers (Rosales and Anseth, 2016; Rizwan et al., 2021). Finally, platforms capable of dynamically altering the surface topography through material erosion, mass migration, or surface wrinkling have also been developed (Zhu et al., 2005; Magin and Anseth, 2013; Rianna et al., 2015).

Despite the wealth of technologies, materials, and manipulation strategies, dynamic platforms often require fairly sophisticated chemical routes and/or specialized equipment to be used. Furthermore, overlapping signals of different natures are usually nontrivial. These observations have limited the development of dynamic platforms beyond the proof-of-concept stage, and nowadays, dynamic platforms are not routinely used for mechanobiology studies. To overcome these limitations, we reported a versatile and rapid method to emboss linear nano-topographies on light-sensitive azobenzene-based substrates on which living cells have previously been confined in circular shapes by means of micropatterning. We studied the variation of cell adhesion, shape, and nuclear configuration over time in response to dynamic topographical stimuli. Cells responded consistently to the changes in surface adhesivity and topography by adapting FAs, cytoskeleton structures, and nuclear matter, ultimately affecting cell mechanics. The ease of material fabrication and functionalization, jointly with the simplicity of implementing the dynamic change of surface adhesivity, makes our approach a valuable route to investigate mechanotransduction-related events *in vitro*.

## Materials and method

### Preparation of the light-responsive substrates

10 mm diameter cover glasses were washed three times in acetone and sonicated for 15 min, and then dried on a heated plate. The spin coating technique was used to produce thin films of Poly-Disperse Red 1-methacrylate (pDR1m) on cover glasses. pDR1m (Sigma-Aldrich) was initially dried overnight and then dissolved in chloroform (Sigma-Aldrich) at a 3% w/v concentration, sonicated, and heated to 37°. 15 µL of this solution was used for each cover glass. The solution was spun over the cover glass using a Laurell spin coater (Laurell Technologies Co.) at 5,000 rpm. The film thickness was assessed with a Dektak 150 Veeco profilometer. Only those samples displaying a uniform coating thickness of (approximately) 300 nm were used for the experimentations and analyses.

### Substrate micropatterning

pDR1m substrates were activated by exposure to a deep UV printer (4D cell, Montreuil, France) for 15 min. Then the samples were incubated for 1 h with 0.1 mg ml^−1^ poly-L-lysine-g-poly (ethylene glycol) (PLL (20)-g [3.5]-PEG (2); SuSoS Surface Technology, Dubendorf, Switzerland) at room temperature (40 µL for each sample). After washing with distilled water, the treated surface was illuminated with deep UV light through a photomask. The quartz photomask consisted of chromium-designed patterns outlining circular islets of 1,300 μm^2^. Before use, the photomask was washed with absolute ethanol and was illuminated for 10 min using the deep UV printer, with its brown side facing the lamp. 2.5 µl of Milli-Q H_2_O (Merck Millipore) was added onto the brown side of the 4D cell quartz mask, and the PEGylated side of the sample was gently dropped onto the mask. Using the deep UV printer, the mask was illuminated for 8 min with the silver side facing the lamp. 5 ml of distilled water was added to the mask to help detach the slides from it. The activated side was then incubated for 1 h at room temperature with 50 µl of fibronectin (Sigma-Aldrich) (final concentration of 50 μg ml^−1^) and then rinsed with PBS. To visualize the protein adsorption on the substrate, we incubated the flat and the micropatterned pDR1m samples with fibronectin, respectively, before and 24 h after the laser inscription. Samples were blocked in 3% bovine serum albumin (Sigma-Aldrich) in PBS for 1 h. Then, samples were stained with a fluorescent anti-fibronectin antibody (dilution 1:100, Sigma-Aldrich) for 1 h at room temperature. Samples were then incubated at room temperature with secondary antibodies (1:300 goat anti-mouse Alexa-Fluor 647). Fluorescent images were collected with an Axio Observer Z1 confocal microscope (Zeiss, Germany). Substrates were excited at 633, and the emissions were collected in the 640–650 nm ranges.

### Cell plating

ASC52telo, hTERT immortalized adipose-derived mesenchymal stem cells were purchased from ATCC. Cells were cultured in a mesenchymal stem cell basal medium (ATCC PCS-500-030). To make the complete growth medium, a mesenchymal stem cell growth kit (ATCC PCS-500-040) for adipose- and umbilical-derived MSCs—low serum components and G418 was added to the base medium. Cells were detached from the culture plate and centrifuged (Thermo Fisher Scientific). Then, cells were resuspended in the culture medium, and 35,000 cells were seeded per substrate. Cultures were performed at 37°C in an incubator with a humidified atmosphere of 95% air and 5% CO_2_. Cell attachment on the substrates was usually observed within 2 h after plating.

### Inscription of the topographic pattern

**FIGURE 1:**
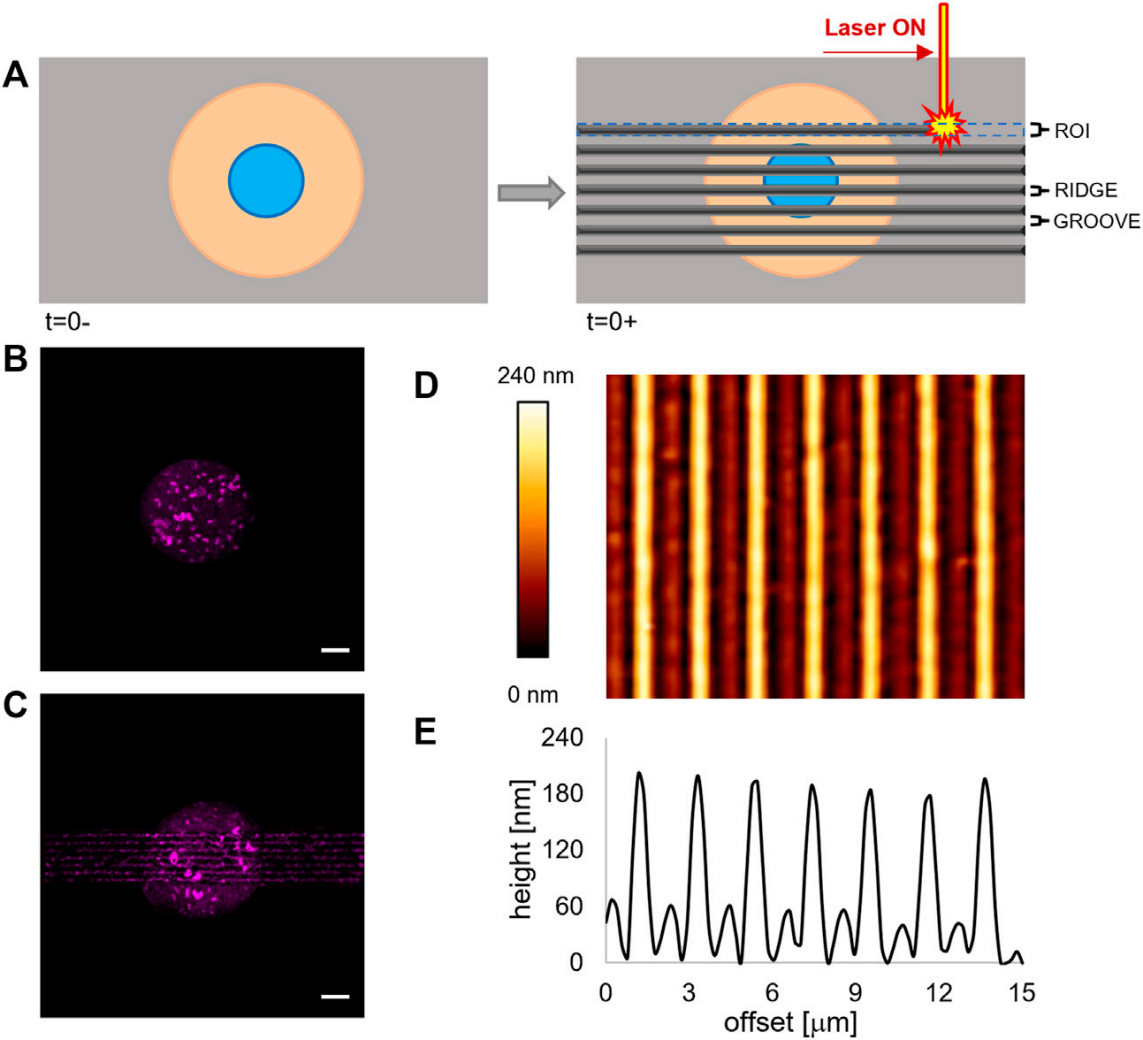
**(A)** Schematic representation of the patterning process *via* laser scanning. **(B)** Fluorescence images of the micropatterned fluorescently labelled fibronectin island on a flat pDR1m substrate prior to pattern inscription and **(C)** on the same substrate after the inscription. Bar is 10 μm. **(D)** AFM image of a pDR1m substrate displaying the laser-written topographic pattern. **(E)** Representative height profile of a horizontal cross section of the topographic relieves.

ACS52telo were cultivated on the fibronectin micropatterned pDR1m coated glass slides for 24 h. Afterwards, real-time pattern inscription on cell-populated samples was performed by using an Axio Observer Z1 confocal microscope (Zeiss). The isomerization of the azopolymer and its consequent mass transport were activated using an Argon laser at 488 nm and 0.44 mW of intensity (measured with a PM100D optical meter (Thorlabs) with a 10 × (N.A. 0.3) objective lens. Patterns were embossed on the pDR1m substrates by illuminating with the laser light in specifically drawn regions of interest (ROIs). Parallel arrays of linear 100 µm long ROIs, separated by 700 nm, were inscribed by placing the circular cell in the centre (Figure 1A). The exposure time was 60 s.

### Atomic force microscopy for characterising substrate surface

An atomic force microscope JPK NanoWizard II (JPK Instruments) was used to acquire images of pDR1m samples. An Axio Observer Z1 microscope (Zeiss) was combined with the AFM to control tips and sample position. Silicon nitride probes (MLCT, Bruker), with a spring constant of 0.01 N/m, were used in contact mode, in air, and at room temperature. Raw images were corrected by the JPK data processing software using a standard procedure (flatten, plane-fit, and artifact lines caused by the tip attachment and removal). The scale indicating the sample height or deflection was adjusted to limit the gap between high and low regions.

### Fixed-cell staining

Cells were fixed at different time-points: 24 h culture on flat substrates, a condition referred to as 0, and 1, 4, and 24 h after pattern inscription. Cell fixation was performed by incubating cells in a 4% paraformaldehyde solution (Thermo Fisher Scientific) for 15 min. Then, cells were permeabilized with 0.1% Triton X-100 (Sigma-Aldrich) in PBS for 15 min. Samples were blocked in 3% bovine serum albumin (Sigma-Aldrich) in PBS for 1 h to avoid non-specific binding. FAs were labelled by incubating samples with an anti-paxillin monoclonal antibody (dilution 1: 400, Sigma-Aldrich) for 1 h at room temperature. Histones were marked by incubating samples with an anti-histone H3 (acetyl K9)—ChIP Grade in monoclonal antibody (dilution 1: 400, Abcam) for 1.30 h at room temperature. After incubation, substrates were washed three times with Triton-PBS (3 min per wash) and incubated with Alexa Fluor 546 conjugated goat anti-mouse antibody (dilution 1: 1,000, Abcam) for 1 h at room temperature. Actin filaments were stained by incubating samples with Alexa Fluor 488 phalloidin (dilution 1: 200, Abcam) for 1 h at room temperature. Finally, cells were incubated for 15 min in DAPI solution (dilution 1:1,000, Abcam) to stain nuclei. Samples were thoroughly rinsed in PBS and placed upside down on the glass. Fluorescent images of FAs and actin bundles were collected with an Axio Observer Z1 confocal microscope (Zeiss). Samples were excited at 488 nm (for phalloidin), 543 nm (for paxillin), and 405 (for DAPI), and the emissions were collected in the 500–530 nm, 560–610 nm, and 420–480 nm ranges, respectively.

### AFM for cell mechanics

Young’s modulus of the living cells cultivated on the substrates was determined by probing the cells with an AFM (JPK NanoWizard II). For this purpose, the probes used were made of silicon nitride cantilevers with a square pyramid tip incorporated at the free end (PNP-DB, NanoWorld, nominal spring constant 0.06 N/m). Spring constants of the cantilevers were measured independently for each experiment. The AFM was mounted on a Zeiss LM 510 confocal microscope to enable AFM tip/cells coalignment. All probe indentations were performed over the central region of the cell in an area where the nucleus was visible. Force curves were typically recorded at a scan rate of 1 Hz, corresponding to a maximum loading rate of 1 nN/s and a maximum force of 1 nN. 64 force curves were acquired at an indentation speed of 1 μm/s over a cell area of 10 × 10 μm. Measurements were performed on each substrate at 24 h of culture on the flat substrate prior to pattern inscription (referred to as 0-), immediately after the inscription of the topographic pattern (termed 0+), and 1, 2, 3, 4, and 24 h after pattern inscription. The number of cells tested varied between 8 and 41 (see Supplementary Material S1). Mechanical properties of cells, in terms of Young’s modulus (E) values, were estimated by fitting the force-indentation curve with the Hertzian model for linearly elastic materials. The individual elasticity values were collected *via* the JPK data processing software providing the distribution of elasticity values per cell. The moduli of the eight highest regions of the cell were considered representative of the elasticity of the nuclear region of each cell. Young’s modulus of the cells was defined as the median of the representative values. To monitor the temporal changes of elasticity, we reported the moduli normalized with respect to the value measured prior to pattern inscription (*t* = 0-).

### Analysis of FAs, cytoskeleton, and nuclear morphology

Information on FA, cell, and nucleus morphology were obtained by analysing digital images of isolated cells at each time point, collected using an Axio Observer Z1 confocal microscope (Zeiss). Analyses were performed using Fiji software, using the command “analyse particles.” This allowed us to extract the values of the area and orientation of the FAs (paxillin channel); area A/R and shape roundness of the cells (phalloidin channel); nucleus A/R and orientation (DAPI channel). The number of analysed cells varied from 6 to 33 (see Supplementary Material S1). Concerning nuclei/FAs orientation on the linear topographic pattern, this was defined as the angle that the principal axis formed with a reference axis, that is, the pattern direction in the case of linear or the horizontal axis for flat surfaces. Nucleus volume was calculated *via* a custom-written script in ImageJ. In brief, Z-stack sections of nuclei were binarized. The area of each section was multiplied by the voxel height (Vh), and the volume was expressed as the sum of the areas × Vh comprising the stack. The (local and global) DAPI average intensity, the H3K9AC average intensity, and the DAPI spot volume were quantified using Imaris software. For Imaris analysis, at least 10 z-stacks per each sample and time point (sections 0.4) were collected (see Supplementary Material S1). The average, median, and standard deviation values of all the parameters listed previously were calculated.

### Statistical analysis

Statistical significance between groups was assessed with either Kruskal–Wallis, followed by a Tukey’s post hoc test, or Mann–Whitman test. Statistical analysis on orientations was performed with Wilcoxon signed-rank test by testing the null hypothesis that the set of measured orientations comes from a distribution with a median of 45°, that is, the median orientation that is expected for a random distribution of orientations. The level of significance was *p* < 0.05.

## Results

### Surface micro- and nanopatterning

In order to regulate FA confinement and cell shape changes, we integrated surface micropatterning with light-responsive materials. Micropatterning produces circular ligand-rich domains (adhesive) surrounded by a cell repulsive PLL-PEG region. The deep UV–based technique we implemented proved to be perfectly compatible with the pDR1m film on which well-defined cell adhesive regions could be formed (Figure 1B).

When irradiated by a laser beam with an adequate wavelength, the azopolymer undergoes a conformational change that results in a microscopic mass migration. We hypothesized that the mass migration would alter the chemistry of the surface by moving the PEG domains and exposing the protein fouling polymethacrylate. To test this hypothesis, we incubated the micropatterned pDR1m sample with fibronectin 24 h after the laser inscription and then stained it with a fluorescent anti-fibronectin antibody. As evident in Figure 1C, both the newly embossed topographic pattern and the circular micropattern are clearly visible. Also, the technique preserves cell viability. In fact, ASC52telo cells cultivated on the circular micropattern for 24 h remained anchored to the substrate after pattern inscription (Supplementary Figure S1). With this information, we embossed a rectangular topographic pattern (100 μm long and 15 μm wide), centred on the circular fibronectin micropattern, constituted by an array of ∼150 nm high ridges separated by ∼1 μm grooves (Figures 1D,E). These dimensions were selected since we observed in previous experiments that they were effective in confining FAs and directing the cytoskeleton assembly (Natale et al., 2014; Iannone et al., 2015). We verified the dimensional stability of the topographic pattern in the culture medium by imaging, with the AFM, the surface of pDR1m substrates, immediately after the inscription or after 6 or 24 h of immersion in the culture medium. The image analyses confirmed that the geometrical features remained unchanged during the 24 h (Supplementary Figure S2).

### Focal adhesion remodelling

**FIGURE 2:**
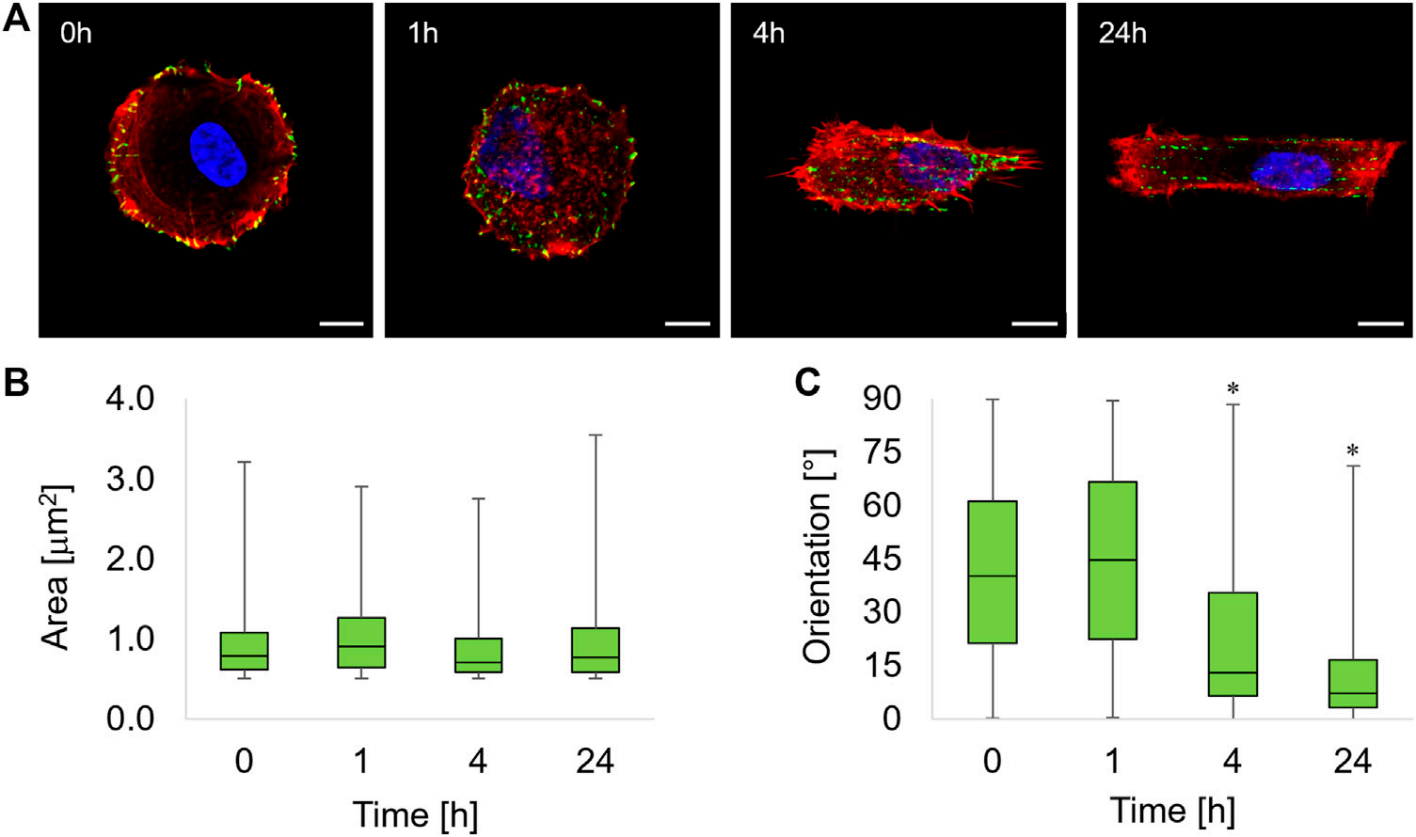
**(A)** Immunofluorescence images of ASC52telo cells cultivated on the flat micropatterned pDR1m substrate (0 h) or on pDR1m displaying the topographic pattern 1, 4, or 24 h after the inscription. Nuclei (DAPI) are in blue, FAs (paxillin) are in green, and actin (phalloidin) is in red. Bar is 10 μm. Box-and-whiskers plot of the FA area **(B)** and FA orientation **(C)**. The asterisks indicate that the median value of the distribution is significantly different from 45°.

To study how cells changed their shape and adapted their morphology following the exposure to topographic signals, we first monitored the dynamics of their anchoring points on the substrate, namely, the FAs. Before embossing the topographic pattern, the whole flat and circular micropattern is available for FA formation. Consistent with other literature reports (Elineni and Gallant, 2011), FAs are mostly located at the pattern periphery, in close proximity to the adhesive/non-adhesive boundary (Figure 2A).

One hour after the inscription of the topographic pattern, a marked destabilization of the FAs was observed, with the presence of small cytoplasmic paxillin spots all over the cell body (Figure 2A, 1h). We also observed FAs starting to coalign with the emerging pattern in the middle third of the cell body, that is, where the topographic pattern crosses the cell. At 4 h post pattern inscription, new FAs formed on the topographic patterns outside the circular adhesive island (Figure 2A, 4h). After 24 h, markedly aligned FAs spots formed within the rectangular topographic patterns, retracing the ridge array (Figure 2A, 24 h). Image analysis revealed that FAs area remained constant within 24 h of the experiment (Figure 2B). The distribution of FA area was right-skewed, with the majority of FAs having an area not exceeding ∼1 μm^2^. To quantify time-dependent changes in the actual adhesive area, we evaluated both the average FA area and the total FA area per cell that were normalized by the basal area of the cell (Supplementary Figure S3). Both parameters attained similar values throughout the experiment, and no significant differences were observed among the different timepoints. The presence of the topographic pattern strongly affected the FA orientation. A marked pattern-FA coalignment was observed at 4 and 24 h as we observed that the median of the distribution of orientation was significantly different from 45° (Wilcoxon test) (Figure 2C).

### Cytoskeleton assembly

**FIGURE 3:**
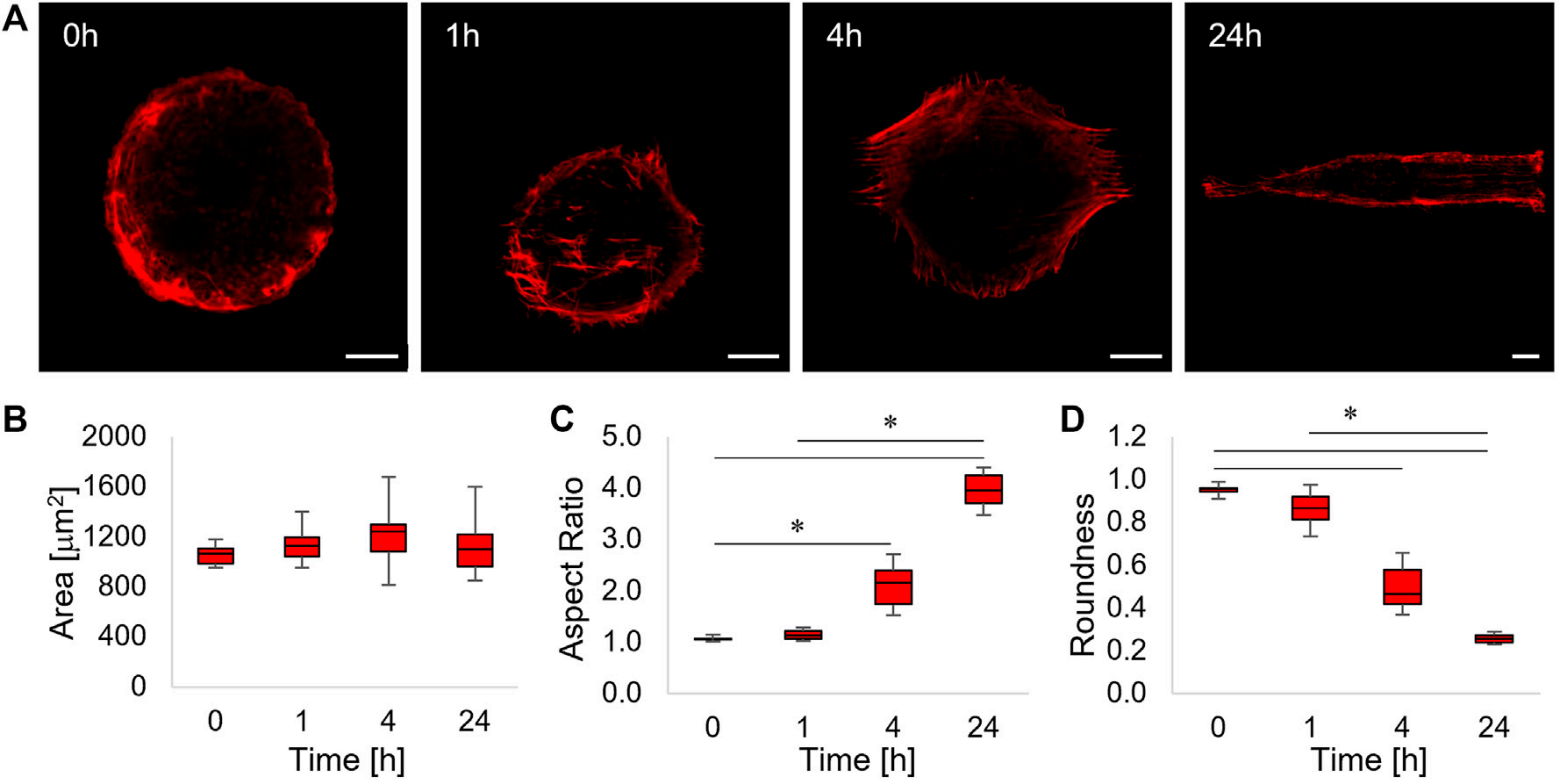
**(A)** Confocal images of phalloidin-stained ASC52telo cells cultivated on the flat micropatterned pDR1m substrate (0 h) or on pDR1m displaying the topographic pattern 1, 4, or 24 h after the inscription. Bar is 10 μm. **(B)** Box-and-whiskers plot of the cell area, **(C)** aspect ratio, and **(D)** roundness. The asterisks indicate statistically significant differences between groups in **(C)**, whereas they indicate that the median value of the distribution is significantly different from 45° in **(D)**.

Changes in positioning and orientation of FAs induce an extensive reconfiguration of the cytoskeleton structure. We acquired confocal images of phalloidin-stained cells at different time intervals. Before the inscription of the topographic pattern (0 h), cells were confined on the circular adhesive microisland displaying an abundance of cortical actin fibres that were mostly circumferentially oriented with a few radial dorsal fibres (Figure 3A).

After 1 h post pattern inscription, a small fraction of actin fibres, located at the centre of the cell body and running parallel to the topographic pattern, were visible (Figure 3A, 1 h). After 4 h, parallel arrays of actin fibres extending outside the circular adhesive island were evident. This trend culminated at 24 h post pattern inscription, in which case cells acquired a rectangular shape and displayed a cytoskeleton constituted by long and parallel fibres extending over the topographic pattern (Figure 3A, 4 h–24 h). In order to get a better insight into the dynamics of cell shape changes, we quantified how cell area, aspect ratio, and roundness changed over time. We found that cell area did not change significantly (Figure 3B). Furthermore, cell’s aspect ratio became significantly higher with respect to the control at 4 and 24 h and significant changes in cell roundness became significant from 4 h onwards, suggesting that the cell’s reaction to the emerging topography requires some time (in the order of a few hours) to be performed (Figures 3C and D).

### Cell shape induced nucleus deformation and chromatin distribution

**FIGURE 4:**
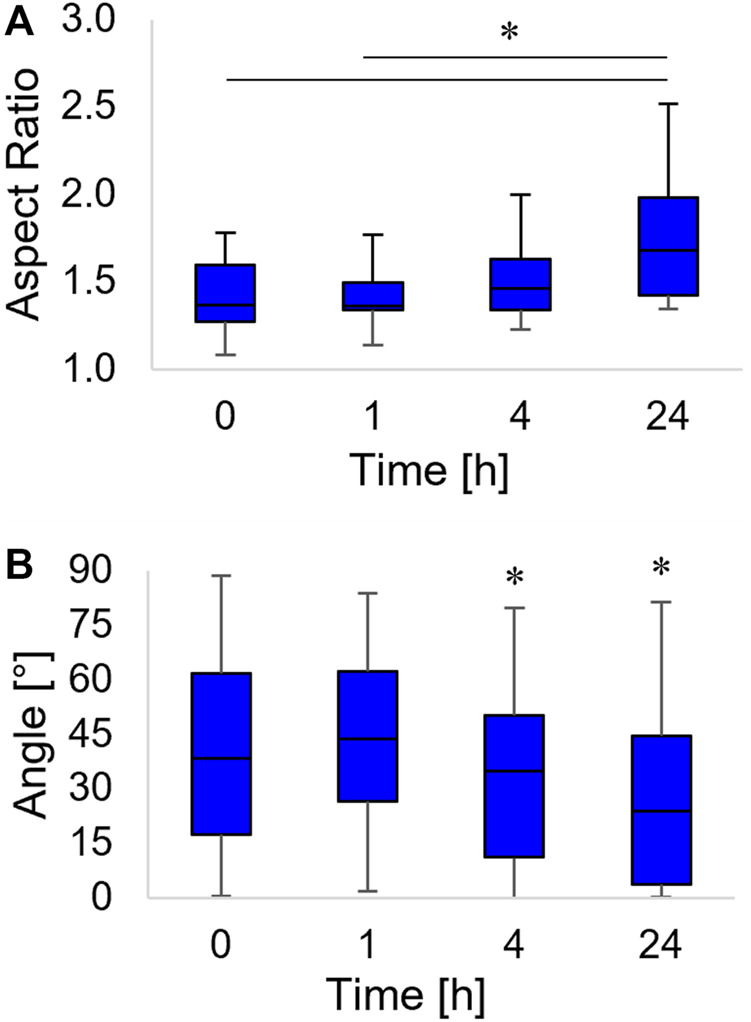
Box-and-whiskers plots of the nucleus aspect ratio **(A)** and the orientation of the nucleus major axis, with respect to the horizontal axis **(B)**. Asterisks indicate significant differences between groups in **(A)**, whereas they indicate that the median value of the distribution is significantly different from 45° in **(B)**.

Changes in the cytoskeleton structure are known to alter the stresses on the nuclear envelope hence affecting nucleus morphology (Natale et al., 2014; Versaevel et al., 2015). We measured how the aspect ratio and the orientation of nuclei evolved in time as the topographic pattern emerged from the pDR1m. We observed a slight, although non-significant, increase of the aspect ratio at 4 h post pattern inscription with respect to time 0; however, the aspect ratio became significantly higher at 24 h (Figure 4A).

The nucleus not only acquired an oblong shape but also rotated along the pattern direction. We observed a coalignment with the pattern direction at 4 h (a time point in which the distribution of the angles of orientation had a median value significantly different from 45°). The nucleus major axis pattern coalignment further increased at 24 h post inscription (Figure 4B).

**FIGURE 5:**
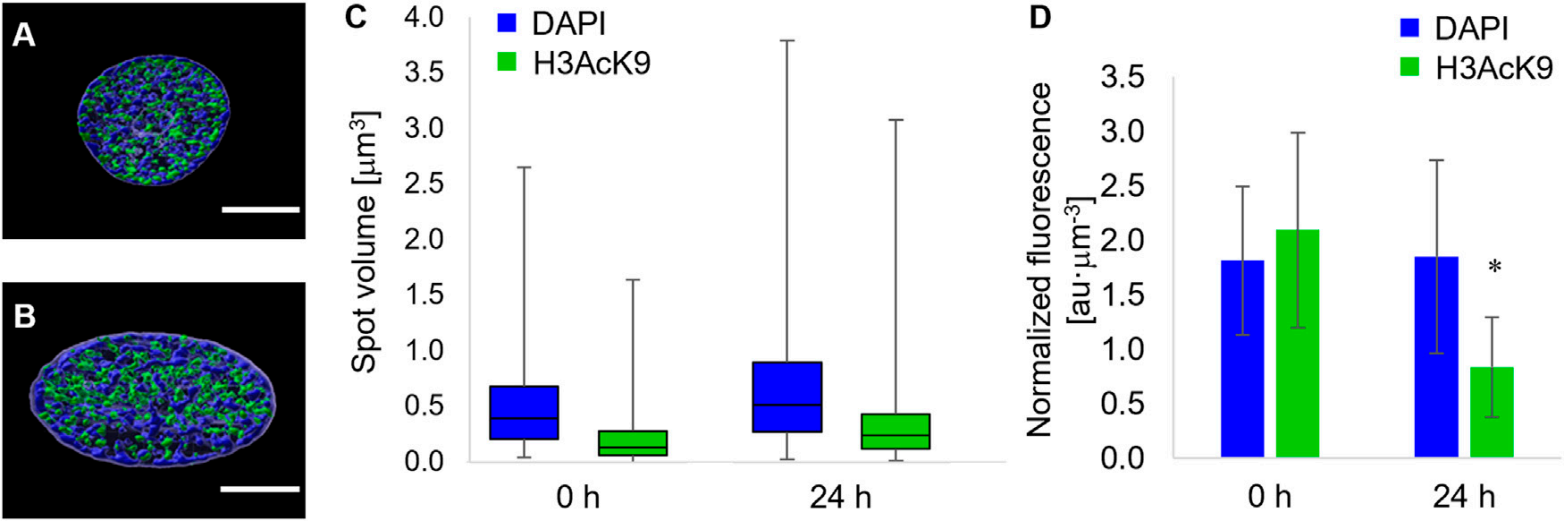
Fluorescence images of equatorial cross sections of nuclei of cells cultivated on **(A)** flat pDR1m substrates or **(B)** 24 h after the inscription of the topographic pattern. DAPI-rich spots are in blue, and H3K9Ac-rich spots are in green. Bar is 5 μm. **(C)** Box-and-whiskers plot of the volumes of the DAPI (blue) or H3K9Ac (green) spots. **(D)** Bar chart of the overall fluorescence intensity of the DAPI or **(D)** H3K9Ac signal. Asterisk indicates a significant difference with respect to the 0 h case.

Forces acting on the nuclear envelope not only alter nuclear morphology but are likely to affect chromatin localization and condensation. In order to quantify the local condensation or decondensation of chromatin, we stained the nuclei of cells with DAPI, and we tagged the acetylated lysine 9 of the histone 3 (H3K9Ac) with fluorescent antibodies. In particular, the uptake of the DAPI stain depends on the total amount of DNA and on its level of condensation. Therefore, the localization of DAPI-positive chromatin domains and their fluorescence intensity are evidence of heterochromatin loci (Görisch et al., 2005). Conversely, the acetylation of histones has been correlated with chromatin decondensation, that is, euchromatin (Görisch et al., 2005; Kouzarides, 2007). Since the most relevant changes in terms of nuclear morphology were observed from time 0 and 24 h post pattern inscription, we limited the study on chromatin localization and condensation to these two timepoints. At the first qualitative observation, the chromatin in the nuclei of cells cultivated on the circular micropatterns, immediately before pattern inscription, was constituted by heterochromatin domains interspersed with H3K9Ac-positive ones (Figure 5A). Interestingly the two domains were sharply disjoined. We could not detect any spatial pattern of either hetero- or euchromatin, although DAPI-positive loci tended to occupy the peripheral part of the nuclei. After 24 h from the inscription of the topographic patterns, the nuclei appeared in the form of oblate ellipsoids in which the DAPI-rich heterochromatin loci were largely present at the inner surface of the nuclear envelope, whereas H3K9Ac-positive domains tended to accumulate within the nuclear core (Figure 5B). In order to assess to what extent chromatin condensation/decondensation occurred, we calculated the distribution of the volumes of the DAPI- and H3K9Ac-positive spots and the global average fluorescence intensity of the DAPI-stained and H3K9Ac-tagged nuclei. The volumes of the DAPI- and H3AcK9-positive spots significantly increased 24 h after the inscription of the topographic pattern (Figure 5C). This trend of volume increase was not followed by an increase in fluorescence intensity. In fact, the overall DAPI fluorescence remained constant in time, whereas the fluorescence signal of the H3AcK9 decreased after 24 h (Figure 5D). Altogether these data are suggestive of local changes in chromatin configuration, with a possible clustering or increase in the dimensions of the heterochromatin domains and an increase in the size of the acetylated histone 3 domains.

### Cell mechanics

The mechanical behaviour of a cell depends on several aspects, including membrane tension, cytoskeleton assembly, and nucleus conformation (Fletcher and Mullins, 2010; Fischer et al., 2020; Sitarska and Diz-Muñoz, 2020). All the structural and morphological changes we have highlighted are expected to affect the mechanical properties of cells. In particular, as the structural and morphological changes occur dynamically, we followed the time-changes of the cell mechanics intended as the stiffness of the nuclear region. We measured the local stiffness of each cell at selected timepoints, and we normalized this stiffness with the stiffness measured prior to pattern inscription. This method enabled us to observe time variations of elastic moduli per cell, not taking into account possible variation in the modulus arising from the intrinsic biological variability among different cells.

**FIGURE 6:**
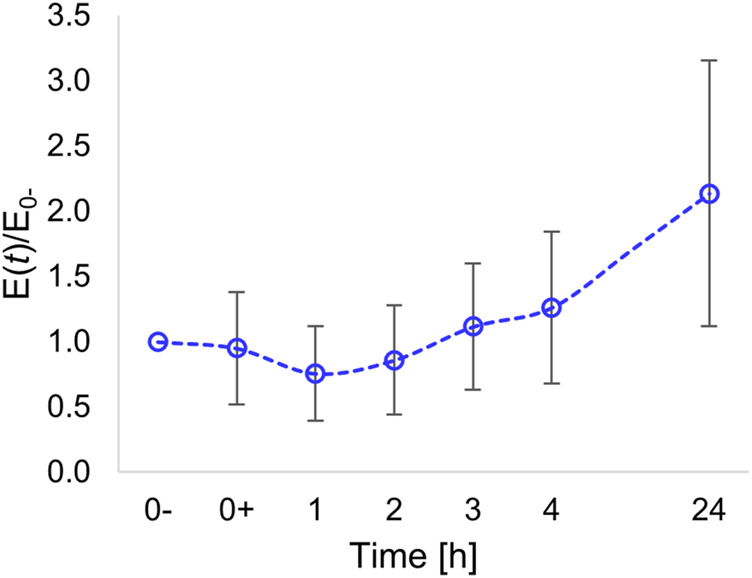
Time variation of Young’s modulus of the nuclear region normalized with respect to the modulus evaluated prior to pattern inscription (at time *t* = 0**-**).

After a slight decrease in the elasticity observed after 1 h, presumably caused by a temporary disassembly of the cytoskeleton (see also Figure 2), the modulus steadily increased (Figure 6).

At 24 h post pattern inscription, we measured a local elastic modulus that, on average, was twice the initial modulus.

## Discussion

Spatiotemporal changes of the composition, architecture, and mechanics of the ECM are fundamentally connected to important biological phenomena (Rozario and DeSimone, 2010; Gattazzo et al., 2014). Cells perceive such a dynamic environment and respond to it by altering their behaviour in terms, for example, of changes in motility, force generation, and gene expression. All these events accompany or trigger the progression of pathologies. The stroma of several cancer types evolves in a way that the collagen network appears straight and densely packed, and these aspects have been suggested to facilitate cell invasion (Egeblad et al., 2010). Also, changes in stiffness and structure of ECM components are a hallmark of fibrosis, in which stiffer fibres in a dysregulated network create an environment amenable to self-sustain cell activation, thus, exacerbating the condition (Liu et al., 2010; Seo et al., 2020). Artificial cell culturing systems replicating certain features of the ECM, such as topography, ligand distribution, or stiffness, have been developed to study or manipulate specific cell functions, including migration and differentiation (Ventre and Netti, 2016a; Xi et al., 2020). However, the majority of these culturing platforms available nowadays are static in nature and, therefore, not adequate to replicate *in vitro* the temporal evolutions that are observed *in vivo*. As mechanosensing and mechanotransduction are intrinsically dynamic phenomena, the fabrication of efficient, dynamically tunable cell culturing platforms would be highly beneficial to systematically studying mechanobiology both in health and disease. Several examples of culturing platforms enabling controlling material stiffness, adhesivity, or topography dynamically have been proposed in the literature. Most of these focus on modulating specific cellular behaviours, such as attachment, migration, and polarization (Cimmino et al., 2018). There are comparatively fewer studies intended to investigate mechanotransduction events through dynamic platforms.

Shape memory polymers or dynamically cross-linkable hydrogels proved to be effective to elicit specific mechanotransduction pathways. For instance, shape memory topographic patterns proved superior capabilities in inducing myogenic differentiation of rat MSCs compared to static substrates (Gong et al., 2014). Also, the chemical/physical properties of these artificial systems can be manipulated in order to program the dynamic changes of the microenvironmental features at different times or length scales. In fact, cells exhibit exquisite mechanotransduction capabilities both at short and long times (Guvendiren and Burdick, 2012). In specific conditions, some cell types promptly restore the initial intracellular localization of mechanoregulating molecules (YAP/TAZ) upon changes in the topography (Mosqueira et al., 2014). In other settings, cells exhibit a persistent mechanical memory, for the initial culturing conditions and the history of exposure to the mechanical inputs of the microenvironment are both crucial in affecting fate decisions. For example, the fate of hMSC depends not only on the stiffness of the substrate cells perceived but also on the past medical history they were exposed to (Yang et al., 2014). More recently, we reported that hMSCs primed on linear gratings, favouring osteogenesis, can be reprogrammed toward other lineages by dynamically changing the topography. However, the re-routing capabilities of the substrates were impaired for longer (14 days) priming (De Martino et al., 2020).

Therefore, cells are able to dynamically sense the biophysical features (mechanical and topographic) and cell functions and fate depend on a complex process of integrating over time the exogenous stimuli and converting them into a biological response.

In this work, we focused our attention on the initial response of hMSC to dynamic changes of the adhesive conditions of the substrate. We opted for light-sensitive polymers as these enable implementing changes of the biophysical features of the microenvironment with a good spatial and temporal resolution (Rianna et al., 2016; Fedele et al., 2020). The light reaches the target immediately and allows to activate/deactivate molecules locally, with a microscale or submicron scale resolution. It is a considerable advantage concerning the other stimuli, such as the temperature, which does not act locally, and the electric fields for which triggering changes of material properties locally could require the fabrication of specifically designed arrays of electrodes. Different from our previous works, here, we combined adhesive micropatterns with a light-responsive surface. This brought in significant improvements as the micropattern generates a more homogeneous population of cells having the same initial shape and similar mechanical properties, whereas the dynamic display of a topography alters the adhesive conditions with a submicrometric resolution. This is beneficial in those experiments in which the initial mechanical conditions of the cell significantly affect the final results, as in the case of studying the cell’s mechanical memory.

The combined technique we illustrated enabled the embossing of a topographic pattern that 1) alters the fouling properties locally, enabling proteins adsorption rapidly, and 2) allows the control of the time-varying mechanical and morphological cell features. Subsequently, for the formation of circular adhesive islands through a photomask, a laser beam of appropriate wavelength and sufficient power from a confocal microscope was employed to modify the protein-repellent coating of PLL-PEG and make the underlying azobenzene substrate adhesive. This technique allowed the manipulation of the adhesive properties of individual cells with submicron resolution, giving rise to topographical features of the desired size in a reasonable time frame, thus, causing alteration in the intracellular architecture in real-time.

After the formation of new adhesive areas, we observed changes in FA positioning and cytoskeleton morphology as early as 1 h after the inscription of the topographic pattern. However, we found significant changes in FA orientation, cell shape, and nucleus orientation after 4 h. The differences became more dramatic after 24 h, a time point at which we also observed a significant change in nuclear shape and cell stiffness. It is likely that the first events that we observed are related to an early remodelling of the FAs that affect cytoskeleton assembly and nuclear orientation. The dynamics of the reconfiguration of FAs are also related to the cell type and its contractile behaviour as cardiomyocytes cultivated on dynamic wrinkles with dimensions comparable to our topographic pattern exhibit an initial (∼4 h) disassembly of paxillin-stained FAs and a subsequent reassembly that restores the original length (Shi et al., 2022).

We did not observe significant changes in FA area, cell area, and FA area to cell area ratio during the 24 h of the experiment. An increase in the total FA area or number of FA per cell is expected to be accompanied by an increase in cell spreading and contractility (Fu et al., 2010; Versaevel et al., 2012). Our data suggest that the modifications to the substrate we imposed promote a reconfiguration of the cell shape rather than an increase in cell spreading that should not significantly alter contractility. Therefore, the observed differences in nuclear shape and cell mechanics are likely to be ascribed to the different assembly of the cytoskeleton that, in cells in an elongated form, is structured in the form of arrays of parallel fibres surrounding the nucleus. This peculiar cytoskeletal structure is much more effective in transmitting compressive forces to the nuclear envelope. This is consistent with the observation of an increased cell stiffness in the nuclear region measured at 24 h, in which case the perinuclear actin cap acts as a fibrous reinforcement. These results agree with experiments reported in the literature performed on static platforms on which highly elongated cells show higher stiffness with respect to round-shaped cells (Kidoaki and Matsuda, 2007). It has to be pointed out that the measurement provides results on the local modulus measured in the nuclear region of each cell. Therefore, the results integrate the contribution of the mechanical properties of different structures constituting the cell, that is, cytoplasm membrane, nuclear membrane, cytoskeleton, nucleoskeleton, and nuclear matter. From such a measurement, it is difficult to discern the contribution of each element to the dynamic changes of the cell modulus. Further experiments involving different but complementary techniques (such as Brillouin spectroscopy) may shed light on the relative contribution of subcellular compartments in dictating the mechanical properties of the nucleus.

In addition to the changes in nuclear architecture and cell mechanics, the changes in the mechanical microenvironment also were effective in altering the euchromatin/heterochromatin stetting in the nucleus. We noticed changes in chromatin configuration in terms of the local redistribution of DAPI-rich or H3K9Ac-rich spots. On a global scale, we also observed a significant decrease in the fluorescence of the H3K9Ac signal, which possibly suggests that cells are undergoing a state of lower transcriptional activity (Qiao et al., 2015). The relationship between chromatin conformation and mechanical stimuli is a central aspect of any mechanobiological setting as nucleus size, volume, or geometry have been implicated in substantial changes in gene transcription (Damodaran et al., 2018). Further experiments should be performed to clarify whether the changes observed in chromatin assembly and acetylation are followed by significant changes in the expression of specific markers for stemness retention or differentiation within the very same time frame. This may also require extending the duration of the experiments, as significant phenotypic changes are observed within days or weeks (Yang et al., 2014; De Martino et al., 2020). The culturing platform presented here provides us with preliminary information on the dynamics of chromatin rearrangements in short timescales (hours). An issue may arise from the particular irradiation wavelength (488 nm) we used in our experiment that can affect cell viability. We did not observe major cytotoxic effects, but this can be partly due to the relatively low intensity we used and the high absorbance of the polymer in the UV range (Ho et al., 1995; Oscurato et al., 2018). However, we demonstrated that topographic inscription might occur at the less phototoxic wavelength of 514 nm, although with shallower relieves (Rianna et al., 2016). Also, supplementing the medium with antioxidants is an additional layer of protection against phototoxicity.

Future improvements in the technique used could help to shed light on different patterns of gene expression obtained in appropriate time intervals. Even though we have reported on the flat-circle to patterned-rectangular transition, different shapes and patterns can be embossed and manipulated over time. For instance, by inscribing a topographic pattern with features depressing FA formation and growth, it is possible to impair stress fibre maturation and contractility. Therefore, an optimization of the laser writing technique to inscribe non-adhesive topographic patterns can be exploited to enable an “on–off” adhesive signal switching locally and at a subcellular scale.

## Conclusion

In this work, we presented a novel optical technique that superposes topographic patterns with submicron scale resolution on biochemically micropatterned azopolymer films. This technique can induce drastic changes in cell morphology, cytoskeleton assemblies, and cell mechanics consequently. We investigated how ASC52telo stem cells reacted to the transition flat-patterned surface in terms of changes in alignment, cell shape, and mechanical properties. Cells’ response was evident from 1 to 4 h post pattern inscription according to the specific event. Changes started from FAs and propagated deep within the chromatin. The reported method enables embossing topographic relief with submicron resolution on bio-functionalized substrates in a few minutes. The substrates thus produced are useful to manipulate cell shape and mechanics in a facile and cost-effective manner and, most importantly, enable to investigate mechanotransduction events dynamically. In particular, the sequential application of biophysical signals can be exploited to study dynamic changes in biological processes, an approach that can be useful to study tissue development, wound healing, and disease progression. Further studies are necessary to clarify the molecular mechanisms that regulate the transduction of material signals in terms of transcription factor flux and the expression of specific genes.

## Data Availability

The original contributions presented in the study are included in the article/Supplementary Material; further inquiries can be directed to the corresponding author.
