# Supplementary material for: A switchable light-responsive azopolymer conjugating protein micropatterns with topography for mechanobiological studies
Source: Front Bioeng Biotechnol. 2022 Jul 22;10:933410. doi: 10.3389/fbioe.2022.933410 (PMC9355574; doi:10.3389/fbioe.2022.933410)
Supplement: Supplementary file 1 [file DataSheet1.PDF]

## SUPPLEMENTARY MATERIAL

# A switchable light-responsive azopolymer conjugating protein micropatterns with topography for mechanobiological studies

**Chiara Cimmino[1,2], Paolo A. Netti1[1,2,3], Maurizio Ventre[1,2,3*]**

[1] Department of Chemical, Materials and Production Engineering, University of Naples Federico II, P.le Tecchio 80, 80125 Naples, Italy

[2] Center for Advanced Biomaterials for Healthcare@CRIB, Fondazione Istituti Italiano di Tecnologia, L.go Barsanti e Matteucci 53, 80125, Naples, Italy

[3] Interdisciplinary Research Centre on Biomaterials, P.le Tecchio 80, 80125 Naples, Italy

* corresponding

**Supplementary Figures**

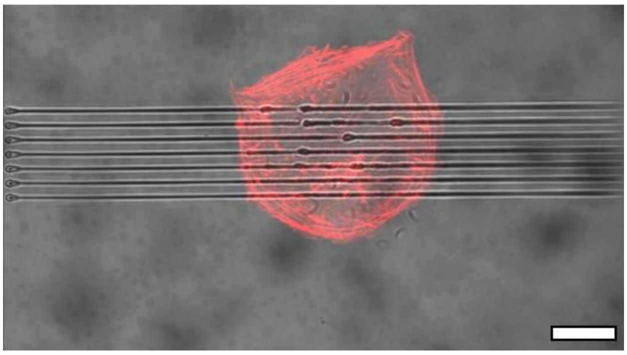

**Supplementary Figure S1.** Confocal images of a single cell after linear pattern inscription (at t=0+). Laser exposure was 60 seconds. Bar 10 μm.

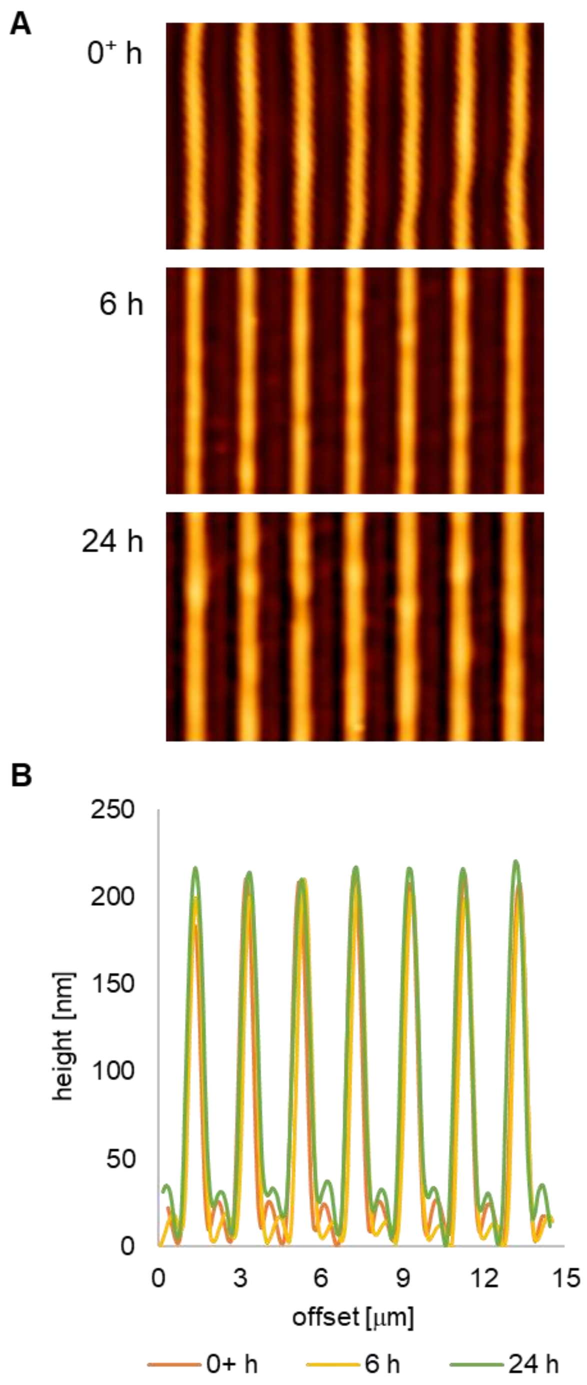


**Supplementary Figure S2.** Dimensional stability of the topographic pattern in culture medium. (A) AFM images of the topographic pattern immediately after the inscription on the pDR1m substrate (0+ h), after 6 and after 24 hours of immersion in culture medium. (B) height profile of three representative horizontal lines of the micrographs in A.

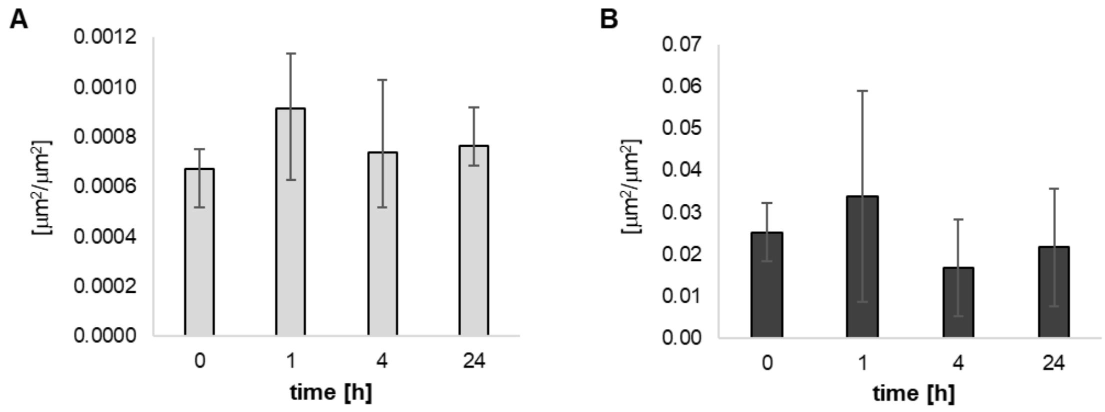


**Supplementary Figure S3**. (A) histogram of the ratios of the average FA area per cell divided by the cell spreading area. (B) histogram of the ratios of the total FA per cell divided by the cell spreading area.

**Supplementary Data**

Here it is reportes a minimal Dataset, in the form of tables, that has been used to construct the charts and graphs in figure 2 – 6.

Focal Adhesion Area (Figure 2B)

| | Time [h] | | | |
|---|---|---|---|---|
| | 0 | 1 | 4 | 24 |
| Minimum | 0.51 | 0.51 | 0.51 | 0.51 |
| Quartile 1 | 0.62 | 0.65 | 0.59 | 0.59 |
| Median | 0.79 | 0.91 | 0.71 | 0.77 |
| Quartile 3 | 1.08 | 1.27 | 1.01 | 1.13 |
| Maximum | 3.22 | 2.91 | 2.75 | 3.55 |
| | | | | |
| Q1-min | 0.12 | 0.14 | 0.08 | 0.08 |
| Q1 | 0.62 | 0.65 | 0.59 | 0.59 |
| Median-Q1 | 0.16 | 0.26 | 0.12 | 0.18 |
| Q3-median | 0.29 | 0.36 | 0.30 | 0.36 |
| Max-Q3 | 2.14 | 1.64 | 1.75 | 2.42 |
| | | | | |
| mean | 0.91 | 1.05 | 0.86 | 0.99 |
| st.dev | 0.41 | 0.52 | 0.42 | 0.59 |
| n FAs | 187 | 211 | 215 | 107 |
| | | | | |
| n cells | 6 | 6 | 10 | 6 |

Focal Adhesion Orientation (Figure 2C)

| | Time [h] | | | |
|---|---|---|---|---|
| | 0 | 1 | 4 | 24 |
| Minimum | 0.06 | 0.28 | 0.05 | 0.03 |
| Quartile 1 | 21.31 | 22.46 | 6.41 | 3.14 |
| Median | 40.08 | 44.72 | 12.98 | 7.18 |
| Quartile 3 | 61.24 | 66.54 | 35.50 | 16.55 |
| Maximum | 89.87 | 89.49 | 88.29 | 71.12 |
| | | | | |
| Q1-min | 21.25 | 22.18 | 6.37 | 3.11 |
| Q1 | 21.31 | 22.46 | 6.41 | 3.14 |
| Median-Q1 | 18.77 | 22.26 | 6.56 | 4.04 |
| Q3-median | 21.16 | 21.82 | 22.53 | 9.37 |
| Max-Q3 | 28.63 | 22.95 | 52.79 | 54.58 |
| | | | | |
| Mean | 41.40 | 45.21 | 23.48 | 10.91 |

| | | | | |
|---|---|---|---|---|
| St.Dev | 25.49 | 26.84 | 23.56 | 11.43 |
| n | 187 | 211 | 215 | 107 |
| | | | | |
| n cells | 6 | 6 | 10 | 6 |

Cell Area (Figure 3B)

| | Time [h] | | | |
|---|---|---|---|---|
| | 0 | 1 | 4 | 24 |
| Minimum | 951.28 | 954.31 | 815.01 | 849.09 |
| Quartile 1 | 987.14 | 1043.53 | 1083.50 | 966.38 |
| Median | 1067.70 | 1127.44 | 1245.08 | 1101.77 |
| Quartile 3 | 1107.06 | 1198.25 | 1300.41 | 1221.45 |
| Maximum | 1180.44 | 1403.96 | 1680.63 | 1601.18 |
| | | | | |
| Q1-min | 35.87 | 89.22 | 268.48 | 117.29 |
| Q1 | 987.14 | 1043.53 | 1083.50 | 966.38 |
| Median-Q1 | 80.56 | 83.91 | 161.58 | 135.38 |
| Q3-median | 39.36 | 70.81 | 55.33 | 119.68 |
| Max-Q3 | 73.38 | 205.70 | 380.23 | 379.73 |
| | | | | |
| mean | 1060.14 | 1139.49 | 1226.58 | 1101.84 |
| st.dev | 80.96 | 133.89 | 256.77 | 206.56 |
| n cell | 13 | 11 | 11 | 16 |

Cell Aspect Ratio (Figure 3C)

| | Time [h] | | | |
|---|---|---|---|---|
| | 0 | 1 | 4 | 24 |
| Minimum | 1.01 | 1.03 | 1.53 | 3.48 |
| Quartile 1 | 1.04 | 1.06 | 1.75 | 3.71 |
| Median | 1.05 | 1.13 | 2.11 | 3.96 |
| Quartile 3 | 1.06 | 1.21 | 2.37 | 4.25 |
| Maximum | 1.15 | 1.29 | 2.71 | 4.40 |
| | | | | |
| Q1-min | 0.03 | 0.04 | 0.23 | 0.23 |
| Q1 | 1.04 | 1.06 | 1.75 | 3.71 |
| Median-Q1 | 0.00 | 0.07 | 0.36 | 0.26 |
| Q3-median | 0.01 | 0.08 | 0.26 | 0.29 |
| Max-Q3 | 0.09 | 0.07 | 0.34 | 0.14 |
| | | | | |
| mean | 1.05 | 1.14 | 2.11 | 3.97 |
| st.dev | 0.04 | 0.09 | 0.40 | 0.32 |
| n cell | 13 | 12 | 11 | 16 |

Cell Roundness (Figure 3D)

| | Time [h] | | | |
|---|---|---|---|---|
| | 0 | 1 | 4 | 24 |
| Minimum | 0.91 | 0.73 | 0.37 | 0.23 |
| Quartile 1 | 0.94 | 0.81 | 0.42 | 0.24 |
| Median | 0.96 | 0.87 | 0.48 | 0.26 |
| Quartile 3 | 0.96 | 0.92 | 0.57 | 0.27 |
| Maximum | 0.99 | 0.98 | 0.66 | 0.29 |
| | | | | |
| Q1-min | 0.03 | 0.08 | 0.05 | 0.01 |
| Q1 | 0.94 | 0.81 | 0.42 | 0.24 |
| Median-Q1 | 0.01 | 0.06 | 0.05 | 0.02 |
| Q3-median | 0.00 | 0.05 | 0.09 | 0.01 |
| Max-Q3 | 0.03 | 0.05 | 0.08 | 0.02 |
| | | | | |
| mean | 0.95 | 0.87 | 0.49 | 0.26 |
| st.dev | 0.02 | 0.08 | 0.10 | 0.02 |
| n cell | 13 | 12 | 11 | 16 |

Nucleus Aspect Ratio (Figure 4A)

| | Time [h] | | | |
|---|---|---|---|---|
| | 0 | 1 | 4 | 24 |
| Minimum | 1.08 | 1.14 | 1.23 | 1.34 |
| Quartile 1 | 1.27 | 1.35 | 1.34 | 1.42 |
| Median | 1.37 | 1.36 | 1.46 | 1.68 |
| Quartile 3 | 1.60 | 1.47 | 1.63 | 1.98 |
| Maximum | 1.78 | 1.77 | 2.00 | 2.52 |
| | | | | |
| Q1-min | 0.19 | 0.22 | 0.11 | 0.08 |
| Q1 | 1.27 | 1.35 | 1.34 | 1.42 |
| Median-Q1 | 0.09 | 0.01 | 0.12 | 0.25 |
| Q3-median | 0.23 | 0.11 | 0.17 | 0.30 |
| Max-Q3 | 0.18 | 0.29 | 0.37 | 0.54 |
| | | | | |
| mean | 1.40 | 1.41 | 1.52 | 1.72 |
| st.dev | 0.21 | 0.15 | 0.24 | 0.30 |
| n cell | 29 | 13 | 28 | 27 |

Nucleus Orientation (Figure 4B)

| | Time [h] | | | |
|---|---|---|---|---|
| | 0 | 1 | 4 | 24 |
| Minimum | 0.35 | 1.78 | 0.05 | 0.12 |
| Quartile 1 | 17.28 | 26.68 | 11.08 | 3.78 |
| Median | 38.51 | 43.70 | 34.99 | 23.93 |
| Quartile 3 | 61.78 | 62.37 | 50.28 | 44.52 |
| Maximum | 88.64 | 83.94 | 79.73 | 81.31 |
| | | | | |
| Q1-min | 16.92 | 24.90 | 11.03 | 3.66 |
| Q1 | 17.28 | 26.68 | 11.08 | 3.78 |
| Median-Q1 | 21.23 | 17.02 | 23.91 | 20.14 |
| Q3-median | 23.27 | 18.67 | 15.29 | 20.60 |
| Max-Q3 | 26.86 | 21.57 | 29.45 | 36.79 |
| | | | | |
| mean | 40.49 | 42.92 | 35.72 | 29.82 |
| st.dev | 27.38 | 27.78 | 25.89 | 28.45 |
| n cell | 33 | 12 | 32 | 25 |

Overall Fluorescence intensity (Figure 5D)

| | DAPI | | H3AcK9 | |
|---|---|---|---|---|
| | 0 h | 24 h | 0 h | 24 h |
| mean | 1.81 | 1.85 | 2.09 | 0.84 |
| st.dev | 0.68 | 0.88 | 0.90 | 0.46 |
| n cell | 10 | 19 | 10 | 19 |

Normalized time variation of cell stiffness $E(t)/E_{0-}$ (Figure 6)

| | Time [h] | | | | | |
|---|---|---|---|---|---|---|
| | 0- | 1 | 2 | 3 | 4 | 24 |
| mean | 0.95 | 0.76 | 0.86 | 1.12 | 1.26 | 2.14 |
| dev.st | 0.43 | 0.36 | 0.42 | 0.48 | 0.58 | 1.02 |
| n cell | 41 | 22 | 16 | 16 | 8 | 21 |
